# Treating AO/OTA 44B lateral malleolar fracture in patients over 50 years of age: periarticular locking plate versus non-locking plate

**DOI:** 10.1186/s13018-020-01622-9

**Published:** 2020-03-20

**Authors:** Chien-An Shih, I-Ming Jou, Pei-Yuan Lee, Chin-Li Lu, Wei-Ren Su, Ming-Long Yeh, Po-Ting Wu

**Affiliations:** 1 Department of Biomedical Engineering, National Cheng Kung University, Tainan, Taiwan; 2 Department of Orthopedics, National Cheng Kung University Hospital, College of Medicine, National Cheng Kung University, Tainan, Taiwan; 3 Department of Orthopedics, National Cheng Kung University Hospital Dou-Liou Branch, Yunlin, Taiwan; 4 Department of Orthopedics, E-Da Hospital, Kaohsiung, Taiwan; 5 School of Medicine, College of Medicine, I-Shou University, Kaohsiung, Taiwan; 6 Department of Orthopedics, Show Chwan Memorial Hospital, Changhua, Taiwan; 7 Institute of Food Safety, National Chung Hsing University, Taichung, Taiwan; 8 Department of Orthopedics, College of Medicine, National Cheng Kung University, Tainan, Taiwan; 9 Medical Innovation Center, National Cheng Kung University, Tainan, Taiwan

**Keywords:** Locking plate, Non-locking plate, Ankle fracture, Fibula fracture, Osteoarthritis, Outcomes

## Abstract

**Background:**

The role of locking plate in lateral malleolar fracture fixation for the elderly remains unclear. The aim of our study is to compare radiological and functional outcomes in older patients (> 50 years) with AO/OTA 44B lateral malleolar fractures after locking plate (PLP) or one-third non-locking tubular plate (TP) lateral fixation.

**Methods:**

We retrospectively reviewed the medical records of 72 patients (PLP group, 34 patients; TP group, 38 patients; mean age, 61.9 ± 7.6 years; range, 51–80 years; follow-up, 1 year). Patients with open fractures, syndesmosis injuries, and a previous ankle trauma or surgery were excluded. Demographic data, union rate, complications, radiographic outcomes, visual analog scale (VAS) scores, and foot and ankle outcome scores (FAOSs) between the groups were recorded and compared. We also investigated the association of clinical features with pain and function. Statistically, the Fisher’s exact test was used for categorical variables and the Mann-Whitney *U* test for the continuous variables. The final model for the multiple regression analysis was used to predict factors related to functional outcomes.

**Results:**

There were no significant between-group differences in demographic data, complication rates, immediately postoperative distal fibula lengths, ankle osteoarthritis (OA) grades, talar tilt angles (TTAs) ≥ 2°, or reduction accuracy. All fractures achieved union. The PLP group had significantly lower rates of distal screw loosening, fibula shortening > 2 mm, OA grade progression, and TTAs ≥ 2°, and better FAOSs and VAS scores than was the case for the TP group after 1 year of follow-up (all *p* < 0.05). The severity of OA, TTA ≥ 2°, and distal screw loosening were positively associated with VAS scores, and negatively associated with FAOSs.

**Conclusions:**

When treating AO/OTA 44B fractures in patients over 50 years of age, PLPs provided better VAS scores, FAOSs, and radiological outcomes, including less fibula shortening > 2 mm, less osteoarthritic (OA) ankle progression, less implant removal rate, and fewer TTAs ≥ 2° than was the case for TPs after a 1-year follow-up.

**Level of evidence:**

Therapeutic level III

## Introduction

The ankle is one of the most common fracture sites in older (> 50 years) people, and more occur with age [1]. Laterally displaced and rotated ankle fractures usually require surgical stabilization [2–4]. A 1-mm displaced talus is associated with more than 40% of tibiotalar contact area decreases and changes [5]. The lateral malleolus is important for ankle mortise stability, especially in AO (Arbeitsgemeinschaft für Osteosynthesefragen )/OTA (Orthopaedic Trauma Association )-44B transsyndesmotic fibula fractures accompanied by mortise changes and talus tilt [6–8].

The Muller technique, using an interfragmentary screw and a non-locking one-third tubular neutralization plate, is recommended for treating AO/OTA 44-B fractures [9]. However, this technique may also lead to fixation failure, further fracture displacement, and poor clinical outcomes in older patients [10]. Open reduction and internal fixation of ankle fractures in older patients may lead to increases in complications [11–13]. Locking plates in distal fibula fractures show superior biomechanical fixation stability in osteoporotic bone [14] and in comminuted artificial bone models [15]. However, clinical outcomes using locking plates for treatment of older patients are still unclear.

Several radiological parameters, including fibular length and talar tilt angle (TTA), have been reported to be related to clinical outcomes in patients with ankle fractures [16–18]. However, perfect radiographs do not guarantee excellent clinical outcomes, where older patients frequently have poorer results [16]. The association of clinical features with pain and functional outcomes in older patients with AO/OTA 44B fractures, the most common type of ankle fracture [19], is still unclear. Therefore, we compared radiological and functional outcomes in older patients with AO/OTA 44B fractures after lateral fixation with either periarticular locking plates (PLPs) or one-third non-locking tubular plates (TPs). We hypothesized that in older patients: (1) PLPs will provide better radiological and functional outcomes than would be the case for TPs and (2) the severity of osteoarthritic (OA) ankle, TTA ≥ 2°, and distal screw loosening will be associated with pain and functional outcomes.

## Materials and methods

### Patients

This is a single-center, analytic, level III, retrospective cohost study. All procedures were approved by our hospital’s Institutional Review Board. Between January 2006 and April 2017, patients over 50 years of age with AO/OTA 44B ankle fractures were treated surgically in our hospital. Our inclusion criteria were patients with an acute AO/OTA type-B transsyndesmotic ankle fracture, age > 50 years [2, 11], surgical fixation with a lateral periarticular locking plate or with a one-third non-locking tubular plate, and the ability to walk without assistance preoperatively. The exclusion criteria were an incomplete radiography or medical record, AO/OTA 44-A or 44-C type fractures, open fractures, fractures with an unstable syndesmosis, a history of previous ankle trauma or surgery, and inadequate follow-up for at least 1 year. Finally, 72 patients who met the criteria were included in this study, including 34 patients in the PLP group and 38 patients in the TP group. The implant type (PLP or TP) was selected by the patient after comprehensive explanations because PLPs are not covered by the national health insurance system. The demographic data of all patients were recorded. Osteoporosis was diagnosed according to previous dual-energy X-ray absorptiometry or a preoperative lateral radiography of the calcaneus as described in previous studies [20–22]. The injury mechanism was classified as *high energy trauma*, e.g., a traffic accident or falling from a substantial height, and *low energy trauma*, e.g., a strain or sports injury. Medical comorbidities such as diabetes, hypertension, and renal disease were also recorded [2].

### Operative technique and post-operative management

**Fig. 1:**
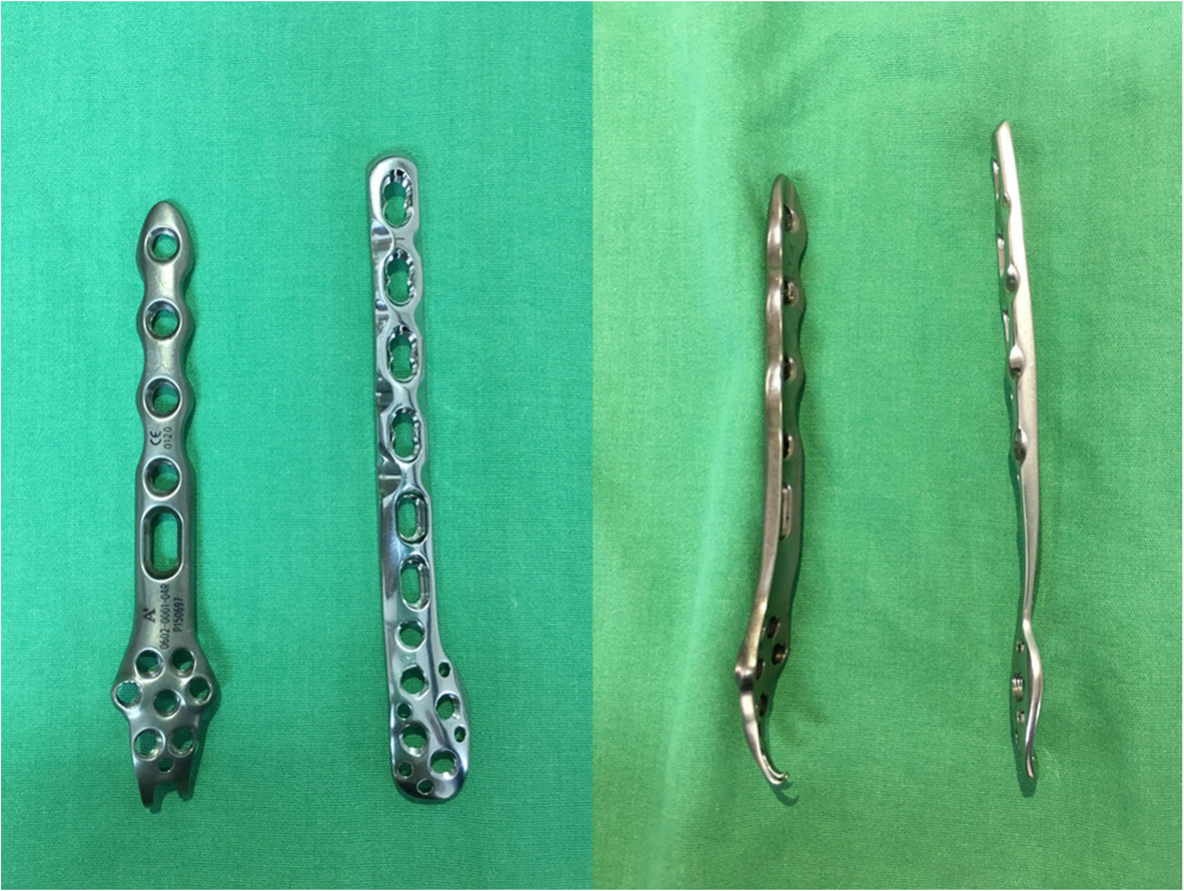
The two low-profile and anatomical periarticular locking plates used in this study

All patients were treated using the same operative technique and postoperative care protocol. Each patient was given a single preoperative and postoperative dose of cefazolin and intravenous (IV) first-generation cephalosporin. A pneumatic tourniquet was used in all procedures, and the lateral malleolar fracture was treated using direct open reduction. A lag screw was placed for interfragmentary fracture fixation when fracture-patterns were suitable, or a reduction clamp was temporarily placed if a lag screw could not be used. Then, a PLP (Fig. 1) (Zimmer Periarticular Distal Lateral Fibular Locking Plate System; Zimmer, Warsaw, IN, USA), a different fibular plate (Distal Fibula Double Hook Locking Plate System; Aplus, Taipei, Taiwan), or a TP (Synthes, Paoli, PA, USA) as a neutralization plate was used for fixation. A medial malleolar fracture was routinely done with one or two screws or augmented K-wires [2] based on the size and type of the fracture. A posterior malleolar fracture was reduced and fixed using one or two 3.5-mm cannulated screws when the involved fragment was more than 20% of the weight-bearing surface. Intraoperative radiographs recorded reduction accuracy and plain films were taken immediately postoperatively to evaluate the reduction. After the index surgery, no weight bearing was allowed for the first 2 weeks and then protected weight bearing was allowed [23]. Full weight-bearing was initiated after 8 weeks or when union was evident radiographically. Splint protection was used for 2 to 4 weeks [2]. Patients were asked to return to our hospital 2 weeks, 1, 2, 3, and 6 months, and 1 year after the operation. If the bony union was not achieved at 6 months after surgery, the patient was asked for an additional follow-up at 9 months. A clinical examination was done, and standard ankle anteroposterior and lateral radiographs were taken serially at each visit.

### Assessing clinical outcomes and measurement parameters

All radiography was reviewed by two senior orthopedic surgeons (WRS and IMJ). When there were disagreements between the two authors, the third author (PYL) joined the discussion until a consensus was reached. The radiological outcomes were evaluated using several parameters. Reduction accuracy was classified as good, fair, or poor according to Lee et al. (good: no fibula shortening, posterior displacement < 2 mm, and < 1 mm increase in the medial clear space; fair: fibula shortening ≤ 2 mm, 2–4 mm posterior displacement, and 1–3 mm increase in the medial clear space; poor: fibula shortening > 2 mm, posterior displacement > 4 mm, and > 3 mm increase in the medial clear space) [2]. Radiologic union was defined as obliteration of the fracture lines or bridging callus across the fracture site on anteroposterior and lateral plain films [23]. The modified Kellgren-Lawrence grade for ankle OA was used to separately evaluate the grade of osteophyte and of joint-space narrowing [24]. The length of the fibular malleolus, the talus tilt angle, and whether the proximal and distal screws were loosening were measured and recorded.

We used foot and ankle outcome scores (FAOSs) [25], which included subscores (score, 0–100; subscales: pain, symptoms, ADLs, sports and recreation [Sports/Rec], and quality of life [QoL]) and total scores (score 0–500) to assess clinical outcomes. Ankle pain was evaluated using the visual analog scale (VAS) (from 0 to 10). Complications were recorded and defined as either wound complications (delayed healing, superficial infection, and deep infection) [26] or other major complications (pulmonary embolism, death, amputation, and revision surgery) [27].

**Fig. 2:**
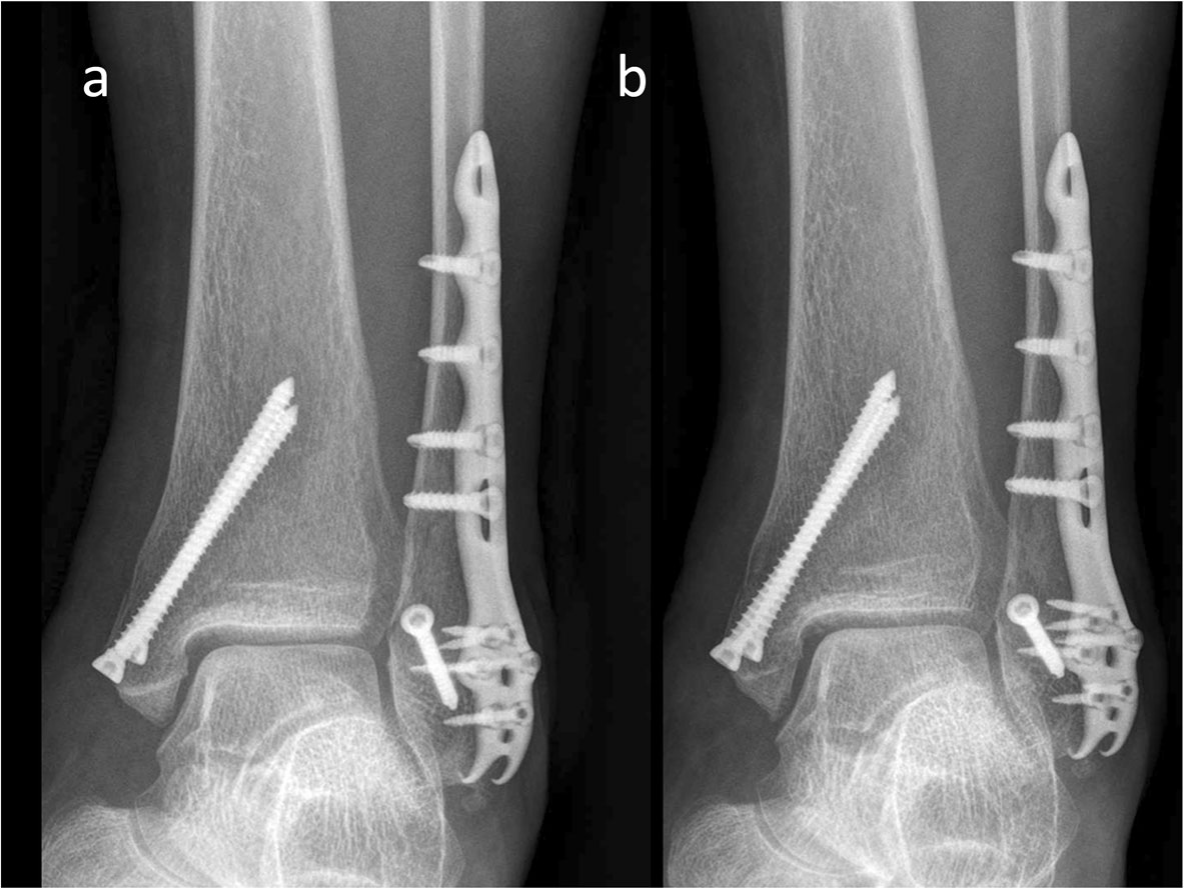
A 64-year-old woman treated using an interfragmentary screw and an Aplus PLP. **a** The fracture was well-reduced (immediately postoperative radiograph). **b** It was united without shortening, further displacement, or changes in OA grade

**Fig. 3:**
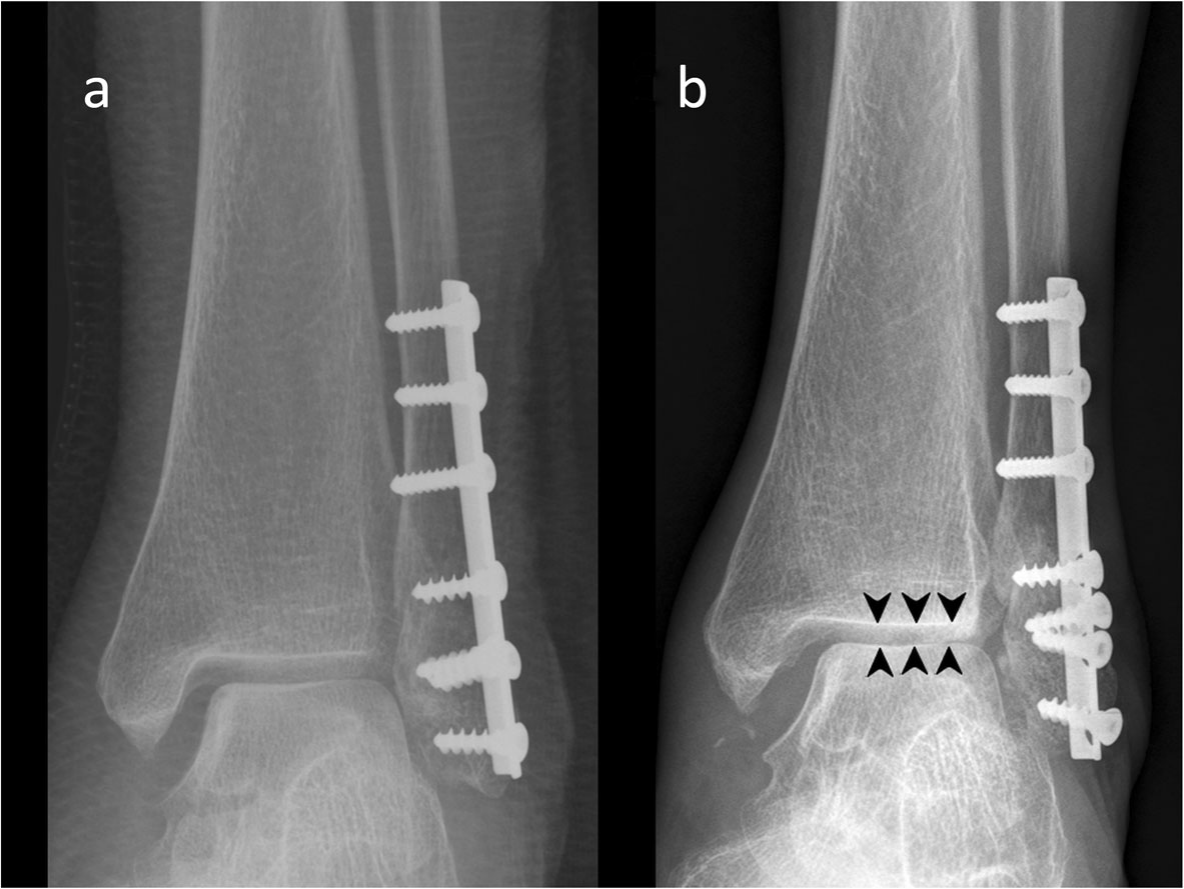
This 78-year-old woman was treated with an interfragmentary screw and a TP. During securing of the interfragmentary screw, the far cortex was cracked, and absolute stability was not achieved. The fibula length could not be anatomically maintained using a TP that was not a fixed angle device. **a** Finally, the reduction accuracy was fair (immediately postoperative radiograph). **b** One year later, it was malunited (the medial ankle-joint substantially widened), the OA grade worsened (arrowheads), and all distal screws were loosened

At the end of the 1-year follow-up, the VAS score, FAOSs, and radiological outcomes (Figs. 2 and 3) of each patient were compared and analyzed with those recorded immediately postoperatively.

### Statistical analysis

Between-group differences in the demographic and clinical characteristics at baseline and the 1-year follow-up were compared. Because of the relatively small sample size in our series, a Fisher’s exact test was used for the categorical variables and the Mann-Whitney *U* test was used for the continuous variables. To investigate the association of clinical features with the functional outcomes, FAOSs, and the VAS pain score, a simple linear regression analysis (Supplementary Table 1) and a multiple linear regression analysis (Supplementary Table 2) were used. The final model for the multiple regression analysis was selected using a forward stepwise procedure and the fewest predictors by which to yield the best interpretations for functional outcomes. SPSS 13.0 (SPSS Inc., Chicago, IL) was used for all statistical analyses. Significance was set at *p* < 0.05.

## Results

### Demographic data and immediate postoperative outcomes

We finally enrolled 72 patients (51 women, 21 men, mean age 61.9 years) with AO/OTA 44 B fractures: 34 patients (24 women, 10 men) were treated with PLP (mean age 63.7 years, range 51–80 years, osteoporosis 24/34) and 38 patients (27 women, 11 men) were treated with TP (mean age 60.2 years, range 51–79 years, osteoporosis 27/38) (Table 1). Except for significantly longer operation time in the PLP group, there were no significant differences in sex, side with lesion, age, proportion of osteoporosis, injury-to-fixation duration, hospitalization duration, injury mechanism, number of malleolus involvements, BMI, comorbidities, initial K-L OA grade, initial distal fibula length, and initial reduction accuracy between the two groups.

**Table 1:** Patient demographics and baseline clinical characteristics

	PLP	TP	*p*
	(*n* = 34)	(*n* = 38)	
Sex			1.000^a^
Male	10	11	
Female	24	27	
Side with lesion			0.815^a^
Left	14	17	
Right	20	21	
Age (years)	63.7 ± 7.9	60.2 ± 7.0	0.065^b^
Operation time (min)	94.7 ± 32.3	80.8 ± 21.7	0.030^b*^
Injury to fixation (days)	1.2 ± 0.6	1.4 ± 1.7	0.453^b^
Hospitalization duration (days)	4.4 ± 2.0	4.0 ± 1.8	0.432^b^
Energy of trauma			1.000^a^
High	21	23	
Low	13	15	
Body mass index (BMI)	26.1 ± 4.0	24.6 ± 2.7	0.086^b^
Fracture type			0.643^a^
Unimalleolar	7	12	
Bimalleolar	18	18	
Trimalleolar	9	8	
Comminuted fracture	2/34	4/38	0.677^a^
Comorbidities			
Diabetes (n)	8/34	5/38	0.359^a^
Hypertension (n)	6/34	4/38	0.501^a^
Renal disease (n)	3/34	3/38	1.000^a^
Osteoporosis	24/34	27/38	1.000^a^
Initial OA grade [24]			0.306^a^
0	0	0	
1	26	24	
2	8	14	
3	0	0	
4	0	0	
Initial talus tilt angle (TTA)			1.000^a^
≥ 2°	2	3	
< 2°	32	35	
Initial distal fibula length (mm)	27.4 ± 3.4	27.3 ± 2.8	0.973^b^
Reduction accuracy [2]			1.000^a^
Good	31	35	
Fair	3	3	
Poor	0	0	

^a^Fisher’s exact test, ^b^Mann-Whitney *U* test, ^*^p < 0.05

### Assessing union, complications, and functional outcomes

All fractures achieved union in both groups. There were no significant between-group differences in time to union (PLP 5.0 ± 1.8 vs. TP 4.9 ± 1.8 months) (Table 2). Wound healing was delayed in two PLP-group patients, and superficial infection was detected in one. In the TP-group patients, wound healing was delayed in two, and a superficial infection was detected in one; however, the implant removal rate was significantly higher in the TP groups. There were no major complications or deep infections, and there were no significant between-group complications. In the PLP group, the 1-year FAOS subscale scores and the total FAOS score were significantly higher, and the VAS score was significantly lower (Table 2).

**Table 2:** One-year clinical and functional outcomes

	PLP	TP	*p*
	(*n* = 34)	(*n* = 38)	
Time to union (months)	5.0 ± 1.8	4.9 ± 1.8	0.742^a^
Union rate [*n* (%)]	34 (100%)	38 (100%)	1.000^b^
FAOS subscale at 1 year			
Pain (0–100)	91.3 ± 7.0	86.1 ± 5.8	< 0.001^a***^
Symptom (0–100)	81.9 ± 9.9	76.7 ± 12.8	0.036^a*^
ADL (0–100)	94.7 ± 4.7	89.6 ± 5.5	< 0.001^a***^
Sports (0–100)	85.6 ± 8.0	80.9 ± 9.9	0.040^a*^
QoL (0–100)	77.6 ± 10.4	70.4 ± 9.8	0.005^a**^
Total scores (0–500)	431.1 ± 31.2	403.7 ± 38.1	0.002^a**^
VAS at 1 year (0–10)	1.2 ± 0.9	2.3 ± 1.2	< 0.001^a***^
Complications			
Delayed wound healing (*n*)	2/34	2/38	1.000^b^
Superficial infection (*n*)	1/34	1/38	1.000^b^
Deep infection (*n*)	0/34	0/38	1.000^b^
Others (*n*)	0/34	0/38	1.000^b^
Implant removal rate	6/34	16/38	0.039^b*^

^a^Mann-Whitney *U* test, ^b^Fisher’s exact test, ^*^p < 0.05, ^**^p < 0.01, ^***^p < 0.001

### Assessing radiological outcomes

Radiological parameters immediately and 1-year postoperatively were significantly different between the two groups: distal fibula length shortening (*p* = 0.002), TTA ≥ 2° (*p* = 0.027), fibula shortening > 2 mm (*p* = 0.018), and distal screw loosening (*p* < 0.001), all after the 1-year follow-up (Table 3). One year postoperatively, OA grades were significantly higher in the TP group (*p* < 0.001) (Table 4).

**Table 3:** Radiographic parameters comparison immediately and 1 year after surgery

	PLP (*n* = 34)			TP (*n* = 38)			*p*
	T0	T1	T1-T0 (ΔT)	T0	T1	T1-T0 (ΔT)	
Distal fibula length (mm) [mean (SD)]	27.0 (3.4)	26.4 (3.0)	1.1 (1.1)	26.8 (3.1)	25.4 (3.0)	1.9 (1.3)	0.002^a,c**^
Talus tilt angle ≥ 2° [*n* (%)]	2 (5.9%)	4 (11.8%)	2 (5.9%)	3 (7.9%)	13 (34.2%)	10 (26.3%)	0.027^b,c*^
Fibula shortening (> 2 mm) [*n* (%)]	0	5 (14.7%)	5 (14.7%)	0	16 (42.1%)	16 (42.1%)	0.018^b,c*^
Proximal screw loosening [*n* (%)]		0			2 (5.3%)		0.495^b,d^
Distal screw loosening [*n* (%)]		0			14 (36.8%)		< 0.001^b,d***^

*T0* immediately after surgery, *T1* 1 year after surgery

^a^Mann-Whitney *U* test, ^b^Fisher’s exact test, ^c^PLP_(ΔT)_ vs. TP_(ΔT)_, ^d^PLP_(T1)_ vs. TP_(T1),_^*^p < 0.05, ^**^p < 0.01, ^***^p < 0.001

**Table 4:** Radiographic OA grade comparison immediately and 1 year after surgery

	PLP (*n* = 34)		TP (*n* = 38)		*p*
	T0	T1	T0	T1	
OA grade [*n* (%)]					
0	0	0	0	0	
1	26 (76.5%)	22 (61.8%)	24 (63.1%)	8 (21.1%)	
2	8 (23.5%)	12 (32.3%)	14 (36.9%)	25 (65.8%)	
3	0	0	0	5 (13.1%)	
4	0	0	0	0	
OA grade progression (T1-T0, ΔT) [*n* (%)]					< 0.001^***^
0		29 (85.3%)		17 (44.7%)	
1		5 (14.7%)		21 (52.6%)	
2		0		0	
3		0		0	
4		0		0	

*T0* immediately after surgery, *T1* 1 year after surgery, ΔT (T1-T0): Changes 1 year after surgery; ^***^p < 0.001 (Fisher’s exact test)

### Association of clinical features with FAOS and VAS scores

In the stepwise multiple linear regression analysis, renal disease, ankle OA grade, distal screw loosening, and TTA ≥ 2° were negatively associated with FAOS scores, but the latter three were positively associated with the VAS score (Table 5).

**Table 5:** Stepwise multiple regression analysis of 1-year FAOS total scores and VAS scores in patients with lateral malleolar fracture (*n* = 72)

	FAOS total score coefficients				VAS coefficients			
	Coefficients (SE)	*t* ratio	a*R*^*2*^	*p*	Coefficients (SE)	*t* ratio	a*R*^*2*^	*p*
Renal disease (yes vs. no)	− 27.24 (12.46)	− 2.84	0.53	0.018^*^	-			
OA grade (0–4)	− 18.56 (6.06)	− 3.22	0.53	0.002^**^	0.52 (0.20)	2.30	0.40	0.011^*^
Distal screw loosening (*n*)	− 15.73 (3.22)	− 4.73	0.53	< 0.001^***^	0.47 (0.11)	3.33	0.40	< 0.001^***^
Talus tilt angle (≥ 2° vs. < 2°)	− 15.79 (7.45)	− 2.12	0.53	0.038^*^	0.53 (0.26)	2.17	0.40	0.043^*^

a*R*^*2*^ adjusted *R* coefficient, *SE* standard error

^*^p < 0.05, ^**^p < 0.01, ^***^p < 0.001

## Discussion

This is the first study to compare the surgical results of AO/OTA 44B fractures treated with lateral PLPs and TPs in patients > 50 years old. There were no significant between-group differences in preoperative demographic data, complication rates, immediately postoperative distal fibula lengths, ankle OA grades, TTAs ≥2°, or reduction accuracy. All fractures achieved union. The TP group had significantly shorter operation times. The PLP group had significantly less fibula shortening, lower rates of distal screw loosening, fibula shortening > 2 mm, ankle OA grade progression, TTAs ≥ 2°, and better FAOSs and VAS scores than was the case for the TP group after 1 year of follow-up. In analyzing the association of clinical features with FAOSs and VAS scores, FAOSs were negatively associated with renal disease, OA grade, distal screw loosening, and TTAs ≥ 2°. The VAS score was significantly positively associated with OA grade, distal screw loosening, and TTAs ≥2°.

Surgical treatment for ankle fracture usually leads to high rates of complication in older people with osteoporosis, inadequate hardware purchase, and poor surrounding soft tissue [2, 10, 13]. Therefore, PLPs have recently become popular for treating osteoporotic lateral malleolar fractures [7, 22]. In OTA 44-B ankle fractures, PLPs are biomechanically superior to conventional non-locking tubular plates in osteoporotic cadaver modes [14, 28], where fixation strength of a PLP is independent of bone mineral density [14, 28]. However, for OTA 44-B and 44-C lateral malleolar fractures, the biomechanical superiority of locked lateral plates cannot be demonstrated when compared with conventional lateral plates in a meta-analysis of biomechanical studies using osteoporotic cadaver models [29]. Up to date, there are several studies evaluating the clinical outcomes in lateral malleolar fractures treated with and without locking plates [3, 4, 13, 22, 23, 26, 30–32] (Supplemental Table 3). However, the reported results vary under the different inclusion criteria for fracture type and implant type. For pure OTA 44-B lateral malleolar fractures, there are two studies in a general patient population [23, 30]. Tsukada et al. [23] reported no significant differences in the 36-Item Short Form Survey (SF-36) score, fibula union rate, and wound complication rate between a locking reconstruction plate group and a non-locking periarticular plate group. Moss et al. [30] reported no difference in the rate of failure and loss of reduction between a PLP group and a non-locking TP group. However, the PLP group had a higher deep infection rate and implant removal rate. For a senior patient population, there are two studies enrolling OTA 44-C type fractures with or without 44-A type fractures in addition to 44-B fractures. Herrera-Perez et al. [22] showed similar average time to union and AOFAS scores in osteoporotic patients aged over 64 using either locking or non-locking TPs. However, time to weight bearing was significantly lower in the locking TP group. Lynde et al. [13] reported that the locking plate group had higher wound dehiscence rates in patients over 60. In our study, we included older patients over 50 years of age and found that the PLP group had significantly less fibula shortening, lower rates of distal screw loosening, fibula shortening > 2 mm, ankle OA grade progression, implant removal, TTAs ≥ 2°, and better FAOSs and VAS scores than did the TP group after 1 year of follow-up. Further studies are necessary to compare the clinical outcomes using locking plates and non-locking plates for AO/OTA 44-B type fracture fixation in older patients.

Fibula length is important for ankle-joint stability, where a loss of lateral malleolar length or alignment may lead to significant biomechanical instability associated with a poor clinical outcome [18, 33]. In our study, the non-locking plate was associated with more fibular shortening, more OA grade progression, and worse FAOS total scores and VAS scores. Other researchers have reported that a shortened fibula is associated with post-traumatic osteoarthritis after fractures, especially in those > 2 mm shorter than the contralateral ankle [18], where there were significantly higher pain scores in post-traumatic OA patients with TTAs ≥ 2° than in those with TTAs < 2° [17]. Our PLP group had less fibular shortening > 2 mm and fewer TTAs ≥ 2° after a 1-year follow-up. This group also had better FAOSs and VAS scores.

Although significant differences were found, treating with a PLP is more expensive. Under our government health insurance system, the cost of a PLP (1800–2000 USDs) is much higher than that of a TP (50–100 USDs). Further studies are necessary to evaluate the cost-effectiveness and clinical indications for PLPs.

Additionally, we found that renal disease, ankle OA grade, distal screw loosening, and TTAs ≥ 2° were negatively associated with FAOSs. Ankle OA grade, distal screw loosening, and TTAs ≥ 2° were positively associated with VAS scores, however. Fibular shortening, TTA, and syndesmotic widening suggest poor outcome, but they are not necessarily significant in all unstable ankle fractures with TTAs ≥ 2° [16]. This may partly explain why our PLP group patients with preserved fibula length had better functional outcomes and pain scale scores and less OA grade progression. We and other researchers [17] found a TTA ≥ 2° to be positively associated with more severe pain scores. In addition, a TTA > 2° is associated with more severe osteochondral lesions [34]. However, fibular length, which has been associated with clinical outcomes [18], was not significantly associated with VAS scores or FAOSs in our study. One possible reason is that the fibular length is correlated with TTA and OA grade. In our multiple regression model, the effect of fibular length was explained by the other two parameters.

Our multiple regression analyses showed renal disease to also be associated with higher pain scores. A possible explanation for this might be that patients with chronic kidney disease tend to have higher prevalence (≥ 70%) of acute and chronic pain [35]. However, the factors that affect pain manifestation in chronic disease are unclear thus far.

## Limitations

This study has some limitations. First, it was a retrospective study, and the choice of implant was left at the discretion of patients depending on their economic or private insurance status. However, to date, the indication for using a locking plate to treat AO/OTA 44B fractures is still unclear. There were no significant between-group differences in the baseline demographic data, and the postoperative differences in radiographic and functional outcome were statistically significant. Second, the methodology used for diagnosis of osteoporosis was mostly confirmed based on preoperatively lateral radiography of the calcaneus, which may have led to bias. However, this method provides an easy, quick preoperative assessment for trauma patients. In a clinical scenario, other quantitative imaging techniques [21] typically are not practical for preoperative assessment. In addition, there were no significant between-group differences in the proportion of osteoporosis. Third, two types of locking plates were used in the PLP group. However, both PLPs provided a low-profile distal structure with more than 4 distal unicortical locking screws for fixation. Fourth, the follow-up was only 1 year; thus, the long-term postoperative radiological and functional outcomes could not be evaluated .

## Conclusions

PLPs used to treat AO/OTA 44B fractures in patients over 50 years of age may provide better FAOSs and VAS scores and better radiological outcomes, less fibula shortening > 2 mm, and OA progression, and fewer TTAs ≥ 2° than TPs after a 1-year follow-up. The severity of OA grade, TTA ≥ 2°, and distal screw loosening were positively associated with VAS scores and negatively associated with FAOSs.

## Supplementary information

**Additional file 1: Table S1.** Simple regression model results for 1-year FAOS total scores and VAS scores in lateral malleolar fracture patients (*n* = 72).

**Additional file 2: Table S2.** Multiple regression analysis for 1-year FAOS total scores and VAS in patients with lateral malleolar fracture (*n* = 72).

**Additional file 3: Table S3.** Literature review on the use of locking plates for lateral malleolar fractures.

## Data Availability

The datasets used during the current study are available from the corresponding author on reasonable request.

## Abbreviations

AO: Arbeitsgemeinschaft für Osteosynthesefragen
OTA: Orthopaedic Trauma Association
PLP: Periarticular locking plate
TP: Tubular plate
VAS: Visual analog scale
FAOSs: Foot and ankle outcome scores
OA: Osteoarthritis
TTAs: Talar tilt angles
