# Supplementary material for: Treating AO/OTA 44B lateral malleolar fracture in patients over 50 years of age: periarticular locking plate versus non-locking plate
Source: J Orthop Surg Res. 2020 Mar 20;15:112. doi: 10.1186/s13018-020-01622-9 (PMC7082938; doi:10.1186/s13018-020-01622-9)
Supplement: Supplementary file 1 — Additional file 1: Table S1. Simple regression model results for 1-year FAOS total scores and VAS scores in lateral malleolar fracture patients (n = 72). [file 13018_2020_1622_MOESM1_ESM.docx]

**Table S1.** Simple regression model results for 1-year FAOS total scores and VAS scores in lateral malleolar fracture patients (n=72)

|  | FAOS total score | | | VAS score | | |
| --- | --- | --- | --- | --- | --- | --- |
|  | Coefficient | (95% CI) | *p* | Coefficient | (95% CI) | *p* |
| Sex (female vs. male) | ‒9.15 | ‒28.17 to 9.88 | 0.349 | 0.20 | ‒0.40 to 0.79 | 0.518 |
| Fracture side (right vs. left) | ‒2.34 | ‒19.91 to 15.23 | 0.795 | ‒0.23 | ‒0.77 to 0.31 | 0.412 |
| Age | ‒0.58 | ‒1.73 to 0.56 | 0.321 | ‒0.02 | ‒0.05 to 0.02 | 0.336 |
| Operation time | ‒0.04 | ‒0.35 to 0.28 | 0.824 | 0 | ‒0.01 to 0.01 | 0.683 |
| Fixation delay (days) | 1.18 | ‒5.46 to 7.83 | 0.728 | ‒0.06 | ‒0.27 to 0.14 | 0.552 |
| Hospitalization days | ‒2.87 | ‒7.48 to 1.74 | 0.227 | ‒0.02 | ‒0.17 to 0.12 | 0.776 |
| Energy of trauma (low vs. high) | 16.32 | ‒1.11 to 33.76 | 0.071 | ‒0.32 | ‒0.87 to 0.23 | 0.259 |
| BMI | 1.78 | ‒0.75 to 4.31 | 0.173 | ‒0.06 | ‒0.14 to 0.02 | 0.125 |
| Fracture type^a^ | ‒6.34 | ‒18.57 to 5.88 | 0.313 | 0.02 | ‒0.36 to 0.40 | 0.931 |
| DM (yes vs. no) | ‒7.32 | ‒29.88 to 15.24 | 0.527 | ‒0.33 | ‒1.02 to 0.37 | 0.362 |
| HTN (yes vs. no) | ‒6.44 | ‒31.56 to 18.67 | 0.617 | ‒0.35 | ‒1.13 to 0.42 | 0.373 |
| Renal disease | ‒42.13 | ‒72.03 to ‒12.23 | 0.007^**^ | 0.21 | ‒0.76 to 1.19 | 0.671 |
| OA grade | ‒34.89 | ‒46.74 to ‒23.05 | < 0.001^***^ | 0.96 | 0.57 to 1.34 | <0.001^***^ |
| Time to union | ‒4.05 | ‒8.95 to 0.85 | 0.110 | 0.14 | ‒0.01 to 0.29 | 0.076 |
| Proximal screw loosening (n) | ‒48.64 | ‒100.36 to 3.07 | 0.070 | 0.71 | ‒0.92 to 2.35 | 0.394 |
| Distal screw loosening (n) | ‒22.46 | ‒29.25 to ‒15.67 | < 0.001^***^ | 0.63 | 0.41 to 0.85 | <0.001^***^ |
| Reduction accuracy | ‒6.09 | ‒37.54 to 25.37 | 0.706 | 0.15 | ‒0.83 to 1.13 | 0.761 |
| Fibula shortening (≥ 2 vs. < 2 mm) | ‒2.52 | ‒40.93 to -4.12 | 0.019^*^ | 0.75 | 0.18 to 1.31 | 0.012^*^ |
| Talus tilt angle (≥ 2° vs. < 2°) | ‒26.74 | ‒46.25 to -7.23 | 0.009^**^ | 0.87 | 0.27 to 1.47 | 0.006^**^ |
| Implant removal (yes vs. no) | 6.508 | ‒12.32 to 25.34 | 0.500 | ‒0.35 | ‒0.92 to 0.23 | 0.247 |

^a^Fracture type: Unimalleolar, bimalleolar, or trimalleolar. ^*^*p* < 0.05,^**^*p* < 0.01, ^***^*p* < 0.001.
