# Supplementary material for: Treating AO/OTA 44B lateral malleolar fracture in patients over 50 years of age: periarticular locking plate versus non-locking plate
Source: J Orthop Surg Res. 2020 Mar 20;15:112. doi: 10.1186/s13018-020-01622-9 (PMC7082938; doi:10.1186/s13018-020-01622-9)
Supplement: Supplementary file 2 — Additional file 2: Table S2. Multiple regression analysis for 1-year FAOS total scores and VAS in patients with lateral malleolar fracture (n = 72). [file 13018_2020_1622_MOESM2_ESM.docx]

**Table S2** Multiple regression analysis for 1-year FAOS total scores and VAS in patients with lateral malleolar fracture (n=72)

|  | FAOS total score coefficients | | | VAS coefficients | | |
| --- | --- | --- | --- | --- | --- | --- |
|  | Coefficients (SE) | 95% CI | *p* | Coefficients (SE) | 95% CI | *p* |
| Renal disease (yes vs. no) | ‒27.28 (11.37) | ‒49.56 to ‒4.99 | 0.019^*^ | - | | |
| OA grade (0-4) | ‒18.59 (5.85) | ‒30.04 to ‒7.12 | 0.002^**^ | 0.51 (0.20) | 0.11 to 0.90 | 0.014^*^ |
| Distal screw loosening (n) | ‒15.77 (3.49) | ‒22.61 to ‒8.92 | < 0.001^***^ | 0.45 (0.12) | 0.21 to 0.68 | <0.001^***^ |
| Talus tilt angle (≥2° vs. < 2°) | ‒15.80 (7.51) | ‒30.51 to ‒1.09 | 0.039^*^ | 0.53 (0.26) | 0.02 to 1.04 | 0.044^*^ |
| Fibula shortening (≥ 2 vs. < 2 mm) | 0.29 (7.31) | ‒14.03 to 14.61 | 0.968 | 0.17 (0.25) | ‒0.33 to 0.66 | 0.513 |

^*^*p* < 0.05,^**^*p* < 0.01, ^***^*p* < 0.001.
