# Supplementary material for: Treating AO/OTA 44B lateral malleolar fracture in patients over 50 years of age: periarticular locking plate versus non-locking plate
Source: J Orthop Surg Res. 2020 Mar 20;15:112. doi: 10.1186/s13018-020-01622-9 (PMC7082938; doi:10.1186/s13018-020-01622-9)
Supplement: Supplementary file 3 — Additional file 3: Table S3. Literature review on the use of locking plates for lateral malleolar fractures. [file 13018_2020_1622_MOESM3_ESM.docx]

**Table S3.** Literature review on the use of locking plates for lateral malleolar fractures

| Study | Year | Study type | AO/OTA type | Patient age | Implant type | Study summary |
| --- | --- | --- | --- | --- | --- | --- |
| Moriarity et al. [3] | 2018 | case series | AO/OTA 44B or 44C | No limitation  (>18 years) | PLPs/NP-LPs  Non-LPs | No differences in complications |
| Herrera-Perez et al. [22] | 2017 | case series | AO/OTA 44B or 44C | >64 years | NP-LPs (TP)  Non-LPs (TP) | No differences in functional and radiologic outcomes |
| Moss et al. [30] | 2017 | case series | AO/OTA 44B | No limitation | PLPs  Non-LPs (TP) | No differences in complications  More cost in PLPs |
| Yeo et al. [4] | 2015 | case series | Not mentioned (including pilon fractures) | No limitation | PLPs only | Easy procedure, providing good patient satisfaction, and achieving complete bony union  Higher risks for metallosis |
| Huang et al. [34] | 2014 | case series | AO/OTA 44A or 44B or 44C | No limitation | PLPs  NP-LPs (MP)  Non-LPs (TP) | PLPs and NP-LPs superior to Non-LPs (functional and radiologic outcomes) |
| Tsukada et al. [23] | 2013 | case series | AO/OTA 44B | No limitation | NP-LPs (RP)  Non-LPs (Periarticular plate) | No difference in functional/radiologic outcomes and complications |
| Kim et al. [35] | 2013 | case series | AO/OTA 44A or 44B | No limitation | NP-LPs (T-shaped locking compression plate) | No soft tissue complication  Implant irritation |
| Lynde et al. [13] | 2012 | case series | No limitation (AO/OTA 44A or 44B or 44C) | >60 years | LPs (type unclear)  Non-LPs (type unclear) | LPs more wound complication |
| Schepers et al. [26] | 2011 | case series | No limitation (AO/OTA 44A or 44B or 44C) | No limitation | NP-LPs (RP and DCP)  Non-LP (TP) | LPs more wound complication |

PLP: Periarticular locking plate; NP-LP: Non-precontouredlocking plate; Non-locking Plate: Non-LP; TP: Tubular plate; RP: Reconstruction plate; DCP: Dynamic compression plate; MP: metaphyseal plate
